# Efficient generation of knock-in transgenic zebrafish carrying reporter/driver genes by CRISPR/Cas9-mediated genome engineering

**DOI:** 10.1038/srep06545

**Published:** 2014-10-08

**Authors:** Yukiko Kimura, Yu Hisano, Atsuo Kawahara, Shin-ichi Higashijima

**Affiliations:** 1 National Institutes of Natural Sciences, Okazaki Institute for Integrative Bioscience, National Institute for Physiological Sciences, Okazaki, Aichi 444-8787, Japan; 2 Laboratory for Cardiovascular Molecular Dynamics, RIKEN Quantitative Biology Center, Furuedai 6-2-3, Suita, Osaka, 565-0874, Japan; 3 Laboratory for Developmental Biology, Center for Medical Education and Sciences, Graduate School of Medical Science, University of Yamanashi, Simigatou 1110, Chuo, Yamanashi, 409-3862, Japan

## Abstract

The type II bacterial CRISPR/Cas9 system is rapidly becoming popular for genome-engineering due to its simplicity, flexibility, and high efficiency. Recently, targeted knock-in of a long DNA fragment via homology-independent DNA repair has been achieved in zebrafish using CRISPR/Cas9 system. This raised the possibility that knock-in transgenic zebrafish could be efficiently generated using CRISPR/Cas9. However, how widely this method can be applied for the targeting integration of foreign genes into endogenous genomic loci is unclear. Here, we report efficient generation of knock-in transgenic zebrafish that have cell-type specific Gal4 or reporter gene expression. A donor plasmid containing a heat-shock promoter was co-injected with a short guide RNA (sgRNA) targeted for genome digestion, a sgRNA targeted for donor plasmid digestion, and Cas9 mRNA. We have succeeded in establishing stable knock-in transgenic fish with several different constructs for 4 genetic loci at a frequency being exceeding 25%. Due to its simplicity, design flexibility, and high efficiency, we propose that CRISPR/Cas9-mediated knock-in will become a standard method for the generation transgenic zebrafish.

Transgenic animals with reporter gene expression in specific tissues or cell types are valuable experimental tools. In zebrafish, an important model organism in developmental and physiological studies, transgenic animals have been generated either by introducing DNA constructs that contain characterized promoter/enhancers^1^,^2^,^3^ or using BAC (bacterial artificial chromosome) mediated methods^4^,^5^,^6^,^7^. However, experimental procedures for both methods are time-consuming steps, involving either characterization of the promoter/enhancer of a gene or the preparation of BAC DNA constructs. Furthermore, it is not always possible to generate transgenic fish that fully recapitulate expression of an endogenous gene even with BAC constructs, because enhancers for gene expression can be located over several hundred kilo-bases away^8^. Consequently, what is lacking in zebrafish is a more convenient and efficient method for knock-in of a large DNA fragment.

Recently, the type II bacterial CRISPR/Cas9 system, a novel RNA-guided nuclease system, has become popular for genome-engineering^9^,^10^,^11^,^12^,^13^. Auer et al.^14^,^15^ have reported that knock-in of a long DNA fragment via homology-independent DNA repair can be achieved in zebrafish using the CRISPR/Cas9 system. In this method, co-injection of a donor plasmid, short guide RNAs (sgRNAs) and Cas9 mRNA leads to concurrent digestion of the genomic DNA and the donor plasmid, resulting in the incorporation of the donor plasmid into the genome. The authors succeeded in converting GFP into Gal4 in two transgenic fish lines with high efficiency^15^. This raised the possibility that knock-in transgenic zebrafish could be efficiently generated using CRISPR/Cas9. However, how widely this approach can be applied remains unclear: no knock-in stable transgenic fish in which foreign genes are integrated into endogenous genomic loci have been established.

Here, we have modified this method, and succeeded in generating knock-in transgenic zebrafish with reporter gene or Gal4 expression that mimics endogenous gene expression for multiple targeted loci. The method is simple, and flexible in design. Furthermore, the efficiency of obtaining transgenic founders is very high (over 25%). We propose that CRISPR/Cas9-mediated knock-in will become a standard method for the generation of transgenic zebrafish lines.

## Results

### Strategy for the generation of knock-in zebrafish with the hsp70 promoter

**Figure 1:**
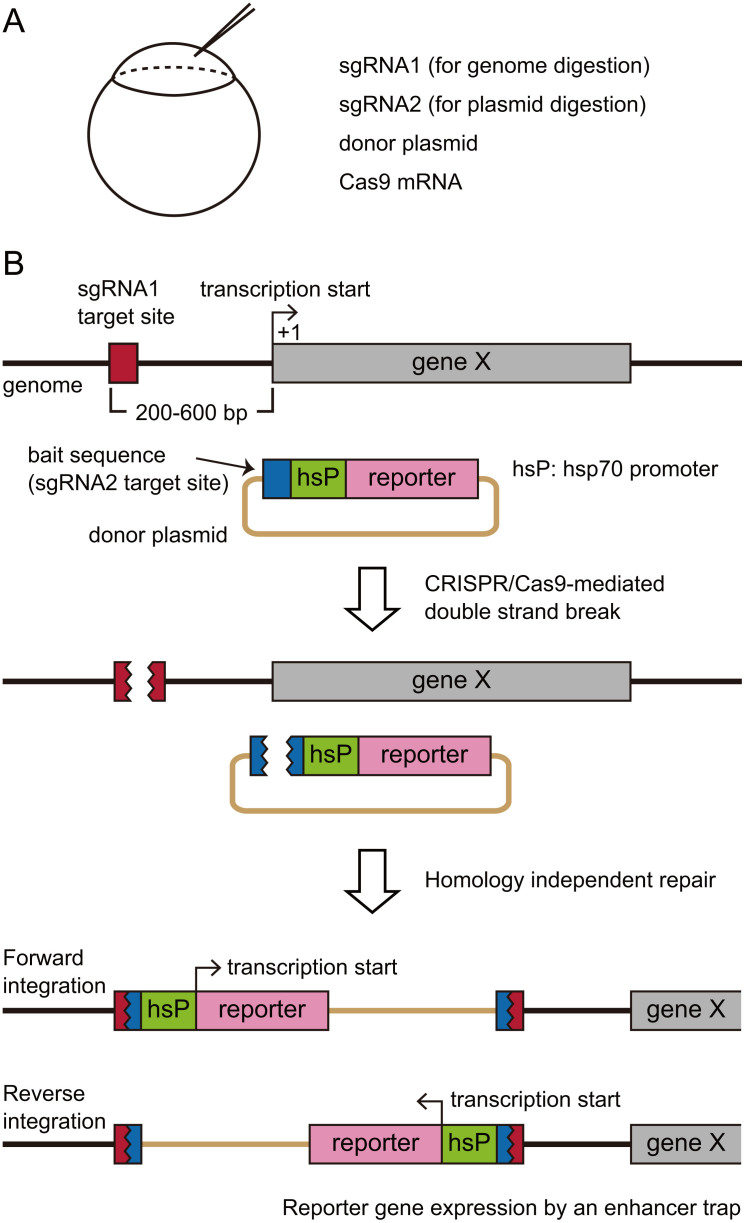
CRISPR/Cas9-mediated knock-in strategy using the hsp70 promoter. (A) For the generation of knock-in transgenic fish, sgRNA1 (for genome digestion), sgRNA2 (for plasmid digestion), the donor plasmid having a bait sequence, and Cas9 mRNA are co-injected into one-cell stage zebrafish embryos. (B) After injection, CRISPR/Cas9-mediated cleavage occurs in the genome at the site upstream (approximately, 200–600 bp) of the gene of interest (gene X). CRISPR/Cas9-mediated cleavage also occurs in the donor plasmid at the bait sequence. This leads to a homology independent DNA repair, resulting in the integration of the donor plasmid into the targeted locus. Both forward and reverse integrations occur. Cis-regulatory DNA sequences for geneX expression act on the hsp70 promoter (enhancer-trapping), resulting in the expression of the reporter gene in cells that express gene X.

The basis of our experimental design followed the one described in Auer et al.^15^: co-injection of sgRNA1 (for genome digestion), sgRNA2 (for plasmid digestion), a donor plasmid, and Cas9 mRNA (Fig. 1A). The donor plasmid contains a bait sequence upstream of the insertion cassette for sgRNA2-guided DNA cleavage. In previous work, a foreign gene that was to be introduced (a modified version of Gal4) was targeted to the coding region of a gene with a 2A peptide linker. With this approach, the inserted gene was transcribed from the promoter that was already present in the genome^15^. Here, we have modified the donor plasmid by introducing the hsp70 promoter (Fig. 1B). sgRNA1 was designed against the genome region that was upstream (approximately, 200–600 bp) of a gene of interest (gene X). Concurrent digestion of the genome (guided by sgRNA1) and the plasmid DNA (guided by sgRNA2) with Cas9 results in the integration of the donor plasmid into the genome via a homology-independent repair system (Fig. 1B). Then, cis-regulatory sequences for tissue-specific expression of gene X act on the hsp70 promoter, resulting in the expression of a reporter gene in cells that express gene X (enhancer-trapping). The primary reason for employing the hsp70 promoter is that it increases the expression level of the transgene, based on our experience of generating BAC-transgenic zebrafish (5, 6). An added benefit of this strategy is that efficiency for the generation of transgenic fish would be increased, as both forward and reverse integrations would yield transgene expression (Fig. 1B).

### Generation of evx2-hs:Gal4 and eng1b-hs:Gal4 transgenic fish

**Figure 2:**
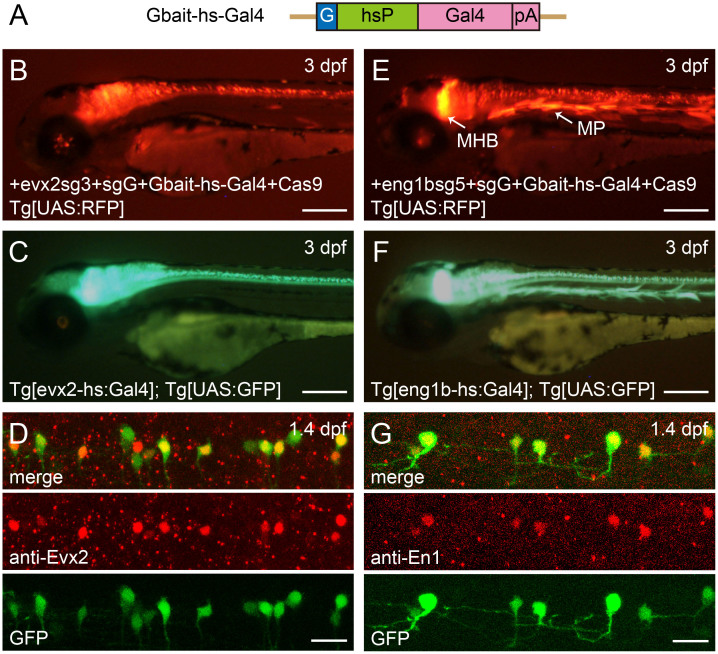
Generation of evx2-hs:Gal4 and eng1b-hs:Gal4 transgenic fish. (A) A schematic of the donor plasmid, Gbait-hs-Gal4. The plasmid consists of Gbait (a bait sequence derived from GFP^15^), hsp70 promoter (hsP), Gal4, and polyA (pA). (B) A 3-dpf Tg[UAS:RFP] embryo showing RFP expression after co-injection of evx2sg3, sgG, the donor plasmid, and Cas9 mRNA. (C) A 3-dpf Tg[evx2-hs:Gal4]; Tg[UAS:GFP] embryo. (D) Spinal cord of a 1.4-dpf Tg[eng1b-hs:Gal4]; Tg[UAS:GFP] embryo was stained with an anti-Evx2 antibody. GFP and Evx2 signals broadly overlap, although some of GFP cells do not have a detectable level of Evx2 signal. (E) A 3-dpf Tg[UAS:RFP] embryo showing RFP expression after co-injection of eng1bsg5, sgG, the donor plasmid, and Cas9 mRNA. RFP is expressed in a subset of neurons in the CNS, cells at the midbrain-hindbrain boundary (MHB), muscle cells possibly derived from muscle pioneers (MP). (F), A 3-dpf Tg[eng1b-hs:Gal4]; Tg[UAS:GFP] embryo. (G) Spinal cord of a 1.4-dpf Tg[eng1b-hs:Gal4]; Tg[UAS:GFP] embryo was stained with an anti-En1 antibody. GFP and En1 signals almost completely overlap. Scale bars: 200 μm in B, C, E, and F; 20 μm in D and G.

We first generated Gal4 transgenic fish for the *evx2* locus. The gene is expressed in subsets of CNS (central nervous system) neurons in embryonic zebrafish^5^. As a bait sequence in the donor plasmid, we used the sequence derived from eGFP (hereafter, called Gbait; Supplementary Fig. S1), as employed by Auer et al.^15^. The donor plasmid consists of Gbait, hsp70 promoter (hsP), Gal4, and polyA (pA) (Fig. 2A). We tested three sgRNAs (evx2sg1, 2 and 3) for genomic DNA digestion (Supplementary Fig. S2). Each sgRNA was co-injected with sgRNA for Gbait (sgG), the donor plasmid, and Cas9 mRNA into Tg[UAS:RFP] embryos. In the case of evx2sg3, some of the injected embryos showed broad RFP expression in CNS cells (Fig. 2B). We raised injected embryos with evx2sg3 to adulthood without pre-selection of RFP expression, and crossed the potential transgenic founders to Tg[UAS:GFP] fish. Among 17 fish screened, two produced embryos with GFP expression that was similar to Evx2 expression (Fig. 2C). For these two lines, insertions of the transgene into the *evx2* locus were verified by PCR. One line had an insertion in the forward direction, while the other had an insertion in the reverse direction. Expression patterns of GFP were similar in both the forward and reverse integration lines (Supplementary Fig. S3A). Auer et al.^15^ reported that in some cases multiple copies of the donor plasmid were integrated within CRISPR/Cas9-mediated knock-in transgenic fish, and thus, more than one copy of donor DNA might have been integrated in the transgenic lines generated here. The direction of the insertion was only examined for the copy that was located at the 5′ end of the integration (this was also the case for the rest of the transgenic fish).

We then tested whether Evx2 expression was recapitulated in the *evx2* transgenic lines. Transgenic embryos fixed and immuno-stained for an anti-Evx2 antibody^5^ demonstrated broadly overlapping patterns of GFP and Evx2 expression, although some of the GFP-positive cells did not have detectable levels of Evx2 (Fig. 2D).

Next, we chose the *eng1b* gene as a candidate for CRISPR/Cas9-mediated knock-in transgenesis. *eng1b* is known to be expressed in a subset of CNS neurons, including cells at the midbrain-hindbrain boundary (MHB), and muscle pioneers (MP)^16^. We initially tried injections with three sgRNAs (eng1bsg1, 2, and 3; Supplementary Fig. S2), as we did for *evx2*, but none of the sgRNA was successful in producing embryos that broadly expressed RFP in the *eng1b* expression domain (more than 50 embryos were examined for each sgRNA). This led us to prepare two additional sgRNAs (eng1bsg4 and 5; Supplementary Fig. S2). We found that injection with the eng1bsg5 was capable of producing embryos that had broad RFP expression in the *eng1b* expression domain (Fig. 2E). We investigated whether there was any correlation between indel-inducing efficiency of each sgRNA and levels of transient expressions. We found that eng1bsg5 had the highest indel-inducing activity (Supplementary Fig. S4), suggesting that the correlation exists.

We raised injected embryos with eng1bsg5 to adulthood without pre-selection of RFP expression, and crossed the potential transgenic founders to Tg[UAS:GFP] fish. Among 40 fish screened, one produced embryos with GFP expression in the *eng1b* expression domain including subsets of CNS cells, cells in the MHB, and MPs (Fig. 2F). PCR analysis showed that the transgene was inserted into the *eng1b* locus with the forward direction. It should be noted that we also obtained one founder fish that produced embryos with GFP expression in cells unrelated to the *eng1b* expression domain (Supplementary Fig. S6). In this fish, the transgene was likely inserted into a locus unrelated to *eng1b*, and an enhancer-trapping occurred.

We then tested whether Eng1b expression was recapitulated in the *eng1b* transgenic line. Transgenic embryos fixed and immuno-stained for an anti-En1 antibody that recognizes Eng1b^17^ demonstrated completely overlapping patterns of GFP and Eng1b expression (Fig. 2G). In summary, Gal4 transgenic fish can be generated with the CRISPR/Cas9-mediated knock-in method. Without pre-selection of fluorescence-positive embryos, the efficiency for the generation of transgenic fish was 12% (2 out of 17) for *evx2*, and 3% (1 out of 40) for *eng1b*.

### High frequency generation of various transgenic fish by pre-selection of fluorescence-positive embryos

**Figure 3:**
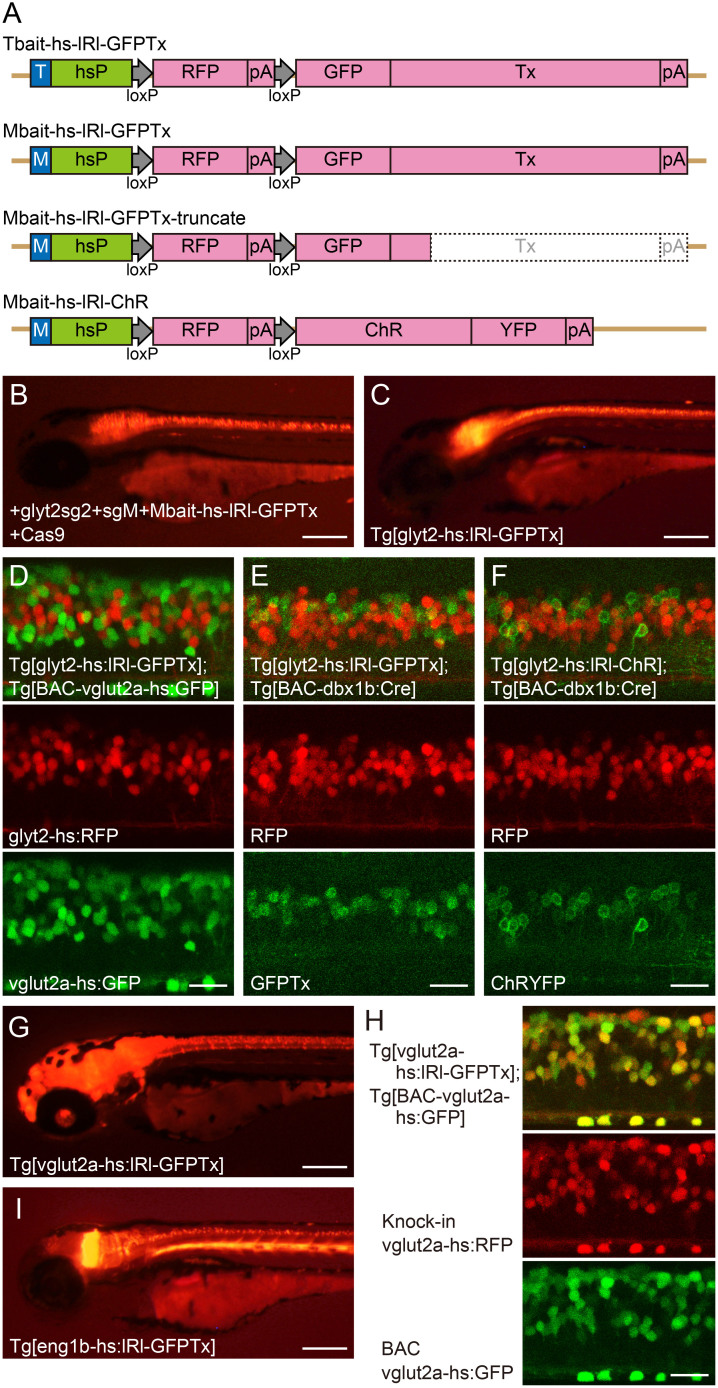
Generation of lRl-GFPTx and lRl-ChR transgenic fish for glyt2, vglut2a, and eng1b loci. (A) Schematics of the donor plasmids having the lRl (loxP-RFP-loxP) expression cassette. The Tbait-hs-lRl-GFPTx and Mbait-hs-lRl-GFPTx plasmids consist of Tbait (a bait sequence derived from Tet1^13^) or Mbait (a bait sequence derived from Mc4r^18^), hsp70 promoter (hsP)^24^, lRl^5^, GFPTx (a fusion construct of GFP and Tetanus-toxin light chain^19^, and polyA (pA). In the Mbait-hs-lRl-GFPTx-truncate plasmid, a part of Tetanus-toxin light chain and polyA are removed from the Mbait-hs-lRl-GFPTx. The Mbait-hs-lRl-ChR has a ChR-YFP fusion construct in the position of GFPTx. (B) A 3-dpf embryo showing RFP expression after co-injection of glyt2sg2, sgM, Mbait-hs-lRl-GFPTx plasmid, and Cas9 mRNA. (C) A 3-dpf Tg[glyt2-hs:lRl-GFPTx] embryo. (D) Spinal cord of a Tg[glyt2-hs:lRl-GFPTx]; Tg[BAC-vglut2a-hs:GFP] embryo at 3 dpf. RFP-positive cells (prospective glycinergic neurons) and GFP-positive cells (prospective glutamatergic neurons) are mutually exclusive. (E) Spinal cord of a Tg[glyt2-hs:lRl-GFPTx]; Tg[BAC-dbx1b-hs:Cre] embryo at 3 dpf. GFPTx is expressed in cells that are likely derived from dbx1b-positive cells. (F) Spinal cord of a Tg[glyt2-hs:lRl-ChR]; Tg[BAC-dbx1b-hs:Cre] embryo at 3 dpf. ChR-YFP is expressed in cells that are likely derived from dbx1b-positive cells. (G) A 3-dpf Tg[vglut2a-hs:lRl-GFPTx] embryo. (H) Spinal cord of a Tg[vglut2a-hs:lRl-GFPTx]; Tg[BAC-vglut2a-hs:GFP] embryo at 3dpf. RFP and GFP cells almost completely overlap. (I) A 3-dpf Tg[eng1b-hs:lRl-GFPTx] embryo. Scale bars: 200 μm in B, C, G, and I; 20 μm in D, E, F and H.

Next, we generated transgenic fish using donor plasmids that contained a GFP sequence. The Gbait was not appropriate as a bait sequence for such constructs, as CRISPR/Cas9 would digest the GFP-coding sequence. Thus, we sought other bait sequences. We wanted to use DNA sequences that are effectively cleaved with CRISPR/Cas9. Previous studies have shown that a 23 bp sequence derived from the mouse *Tet1* gene (called Tbait, hereafter) and a 23 bp sequence derived from the rat *Mc4r* gene (called Mbait, hereafter) are effectively cleaved by CRISPR/Cas9^13^,^18^. Thus, we employed these two sequences as baits (Supplementary Fig. S1). We first constructed two donor plasmids, Tbait-hs-lRl-GFPTx and Mbait-hs-lRl-GFPTx (Fig. 3A). These plasmids contained a bait (Tbait or Mbait), the hsp70 promoter (hsP), loxP, RFP, polyA (pA), loxP, GFPTx, and polyA (pA). GFPTx is the fusion construct of GFP and Tetanus-toxin light chain, which blocks neuronal transmission^19^. As a target locus, we chose *glyt2*, which is known to be expressed in prospective glycinergic neurons in the CNS^20^. We prepared three sgRNAs (glyt2sg1, 2, and 3), and each sgRNA was co-injected with sgRNA for Tbait or Mbait (sgT or sgM), the donor plasmid, and Cas9 mRNA into wild-type embryos. When we injected glyt2sg2, approximately 5–10% of embryos expressed RFP broadly in the *glyt2* expression domain^20^ both for the Tbait and Mbait plasmid donors. Figure 3B shows an example of the Mbait plasmid.

To obtain stable transgenic fish, we raised the injected embryos to adulthood. To examine whether there was any correlation between the levels of transient expression of RFP and transgene integration events in the germline, we separated the embryos into those having broad RFP expression (approximately, 5–10% of the injected embryos) and those with poor or no RFP expression (an example of the screening is shown in Supplementary Fig. S7). For the former (good expression in transients), we obtained 6 positive founders from 10 potential founders (4 out of 7 for Tbait; 2 out of 3 for Mbait). For the latter (poor or no expression in transients), no positive founders were obtained from 27 potential founders (15 for Tbait and 12 for Mbait). Thus, there was a very strong correlation between the expression level of the transgene in transients and the frequency of integration events in the germline.

Expression patterns of RFP in the 6 transgenic lines were all similar. An example is shown in Figure 3C. As seen in the evx2hs:Gal4 transgenic fish, both forward and reverse integrations were observed (Supplementary Table S1). RFP expression levels in the reverse integration line were weaker than the forward integration lines (Supplementary Fig. S3B).

To confirm RFP expression in glycinergic neurons in the transgenic fish, we crossed the glyt2-hs:lRl-GFPTx transgenic fish with Tg[BAC-vglut2a-hs:GFP] in which GFP is expressed in prospective glutamatergic neurons^5^. As expected, RFP-positive neurons and GFP-positive cells were mutually exclusive (Fig. 3D). The lRl-GFPTx construct was designed to enable GFPTx expression in Cre-expressing cells. To verify this, we crossed the glyt2-hs:lRl-GFPTx transgenic fish with Tg[BAC-dbx1b-hs:Cre] transgenic fish^5^. In 4 of the 4 lines tested, GFPTx expression was observed in a subset of spinal neurons that were likely derived from *dbx1b*-positive cells (Fig. 3E), verifying Cre-dependent GFPTx expression. The compound transgenic fish of Tg[glyt2-hs:lRl-GFPTx] and Tg[BAC-dbx1b-hs:Cre] did not show an apparent behavioral phenotype, suggesting that expression levels of GFPTx was not high enough to completely block the neuronal transmission in the GFPTx-expressing cells.

We further generated *glyt2* transgenic fish with two other constructs. One was Mbait-hs-lRl-ChR (ChR represents channel-rhodopsin, a light-dependent cation channel^21^), and the other was Mbait-hs-lRl-GFPTx-truncate (the latter was a mistake product during the construction process of Mbait-hs-lRl-GFP; see Methods). For these two donor plasmids, we only raised embryos that had broad RFP in the *glyt2* expression domain (approximately, 5–10% of the injected embryos). One positive founder was obtained from 5 fish for the Mbait-hs-lRl-ChR, and 7 positive founders were obtained from 14 fish for the Mbait-hs-lRl-GFPTx-truncate (for the direction of the integration, see Supplementary Table S1). These results further confirmed the high-frequency generation (8 out of 19; 42%) of knock-in transgenic fish using the pre-selection process. All the transgenic embryos showed a similar RFP expression pattern in prospective glycinergic neurons, although expression levels of RFP tended to be weaker in the lines with reverse integrations. In the Tg[glyt2-hs:lRl-ChR] transgenic fish, Cre-dependent expression of ChR (YFP fusion protein) was verified by crossing the line with Tg[BAC-dbx1b-hs:Cre] (Fig. 3F).

Finally, we generated transgenic fish for two more loci, *vglut2a* and *eng1b*, with the donor plasmid Mbait-hs-lRl-GFPTx. For *vglut2a*, two sgRNAs, vglut2asg1 and vglut2asg2, were prepared. We found that injections with the vglut2asg2 yielded embryos with broad RFP expression in the *vglut2a* expression domain (approximately, 5–10% of the injected embryos). We raised those embryos to adulthood. Four positive founders were obtained from 15 screened fish (27% in efficiency). All the transgenic fish had forward integrations of the transgene, and the expression patterns of RFP were all similar. An example is shown in Figure 3G. We crossed one of the Tg[vglut2a-hs:lRl-GFPTx] fish with Tg[BAC-vglut2a-hs:GFP] fish^5^. As expected, RFP-positive cells and GFP-positive cells almost completely overlapped (Fig. 3H).

Generation of Tg[eng1b-hs:lRl-GFPTx] lines was performed with eng1bsg5, the same sgRNA used for the generation of Tg[eng1b-hs:Gal4]. We raised injected embryos that had broad RFP expression in the *eng1b* expression domain (approximately, 5–10% of the injected embryos). Three positive founders were obtained from 10 screened fish (30% in efficiency). Two had forward integrations, and one had a reverse integration. Expression patterns of RFP were all similar (Supplementary Fig. S3C). An example is shown in Figure 3I. Taken together, the above results indicate that knock-in transgenic fish can be generated with an efficiency exceeding 25% with the pre-screening process of transgene expression.

## Discussion

### Efficient generation of transgenic zebrafish with CRISPR/Cas9

Here, we have shown that knock-in transgenic zebrafish with Gal4 or reporter gene expression can be efficiently generated by performing co-injection of two sgRNAs (one for digestion of the genome and the other for the digestion of the donor plasmid), the donor plasmid, and Cas9 mRNA. This is the first report for the generation of stable transgenic zebrafish in which large DNA fragments were knocked-in into endogenous genomic loci using CRISPR/Cas9. The procedures are simple and reliable. A critical step is the preparation of a sgRNA that can yield embryos with a broad expression of fluorescent protein in the expression domain of the target gene. In most cases, testing three sgRNAs is sufficient, although in some cases (eg, *eng1b*), preparation of more sgRNAs may be required. Once effective sgRNAs are identified, raising embryos that have broad fluorescent protein expression can yield transgenic founders with an efficiency exceeding 25% (54% for *glyt2*, 27% for *vglut2a*, and 30% for *eng1b*). It is likely that similar efficiency can be achieved in any genetic loci.

Expression levels of fluorescent protein in injected embryos depended on sgRNAs: some lead to good expression, while others lead to poor expression. This is likely to be caused by the cleavage efficiency of the genomic DNA with CRISPR/Cas9^10^. Indeed, in the case of sgRNAs for the *eng1b* gene, the sgRNA that showed the best results in the transient expression assay (eng1bsg5) showed the highest indel-inducing efficiency (Supplementary Fig. S4) The sgRNAs used for the generation of transgenic fish for the other three genes also had an indel-inducing capability (Supplementary Fig. S5). Efficiency of DNA cleavage (indel-inducing efficiency) has generally been measured with PCR after preparation of genomic DNA. With our approach, good sgRNAs can be selected by just observing fluorescent protein expression levels in injected embryos.

There was a strong correlation between expression levels of fluorescent protein in transients and integration events in the germline. With the Tbait- and Mbait-hs-lRl-GFPTx constructs, the animals that showed broad RFP expression in transients became positive founders with a frequency of 60% (6 out of 10). In contrast none of the animals that showed poor or no RFP expression became positive founders (n = 27). Thus, it is critical to raise only those embryos that show good expression. Frequency of the appearance of such embryos among injected embryos was approximately 5–10% for all three genes tested. This low frequency, however, is not an obstacle, since injections can be routinely performed in a large number of embryos (>100). Instead, raising animals to adulthood and performing pair crossing are more time-consuming steps for the generation of transgenic fish.

The basic method used in Auer et al.^15^ and this study (concurrent injection of sgRNAs and donor plasmid) can be applied not only for the generation of simple reporter/driver lines, but also for performing more sophisticated genome modifications such as tagging of open reading frames with fluorescent protein. In this case, the frequency of obtaining embryos that show good expression is expected to be much lower than the present study, since an in-frame fusion with a forward integration is required. Nonetheless, a strong correlation between fluorescent protein expression in transient and integration events in the germline would make the experiment feasible: It would be critical to inject a large number of embryos and select only those that have broad expression of fluorescent protein.

The three bait sequences, Gbait, Tbait, and Mbait all worked well in the present study. Among them, Gbait is not suitable as a bait for donor plasmids that contain GFP sequences. By contrast, Tbait and Mbait sequences, derived from rodent genomes, are unlikely to be present in donor plasmids that are to be introduced. In the zebrafish genome, we identified no potential off-target binding sites of sgM and sgT with up to two mismatches (Supplementary Table S2). Thus, Tbait and Mbait would be useful for any construct used to make transgenic zebrafish.

### The usage of the hsp70 promoter

Instead of knocking-in DNA constructs in the exon of a gene of interest, we knocked in constructs in the upstream region of a gene with the hsp70 promoter construct (Fig. 1B). There are advantages and a potential disadvantage in this approach. An advantage is that expression levels of transgenes are increased. In our experience generating BAC-transgenic zebrafish, the usage of the hsp70 promoter instead of promoters of endogenous gene promoters increased the expression level of the transgene for many genes including *glyt2*, *vglut2a*, *dbx1b*, *gata3*, and *dmrt3a*. Another advantage is that a relatively large genomic region can be a subject of integration. This allows for tests of many sgRNAs. If promoter-less constructs are desired, the primary target site is the 5′-leader sequence (between the transcription start site and the initiation ATG). This region might be short in some genes, and there might be few candidate target sites (the target sequence must contains GG^11^). A potential disadvantage is that gene expression may not be completely recapitulated with the usage of a heterologous promoter. In Tg[evx2hs:Gal4]; Tg[UAS:GFP] transgenic fish, some of the GFP-positive cells did not express detectable level of Evx2 (Fig. 2D). This could be due to leaky expression caused by the usage of the hsp70 promoter. If this kind of leakiness represents a problem, promoter-less DNA constructs must be used.

We should mention that for some purposes, expression levels of transgenes may not be sufficient even with the hsp70 promoter. For example, expression levels of GFPTx in the *glyt2* transgenic fish appeared not strong enough to completely block neuronal transmission in the expressing cells. In order to achieve extremely high levels of transgene expression, binary expression systems such the Gal4-UAS system seem to be required.

### BAC-transgenics vs. CRISPR/Cas9-mediated knock-in

Until now, BAC-mediated transgenics has been the standard for the generation of transgenic fish. CRISPR/Cas9-mediated knock-in has several advantages over BAC-transgenics. The main advantage is that the construction process with CRISPR/Cas9 is by far easier and less time-consuming. In addition, the frequency of obtaining positive founders is higher (over 25% in CRISPR/Cas9 in this study vs. around 10% with BACs in our previous studies), as long as embryos having good transgene expression are raised. Finally, for some genes, it can be difficult to obtain good transgenic lines with BACs: enhancers for gene expression may be located far away from the gene. Indeed, in our previous attempt to generate *eng1b* BAC transgenic fish, a genomic sequence in a BAC that covered 60 kb upstream and 60 kb downstream was insufficient for the full recapitulation of reporter gene expression in *eng1b*-positive cells (our unpublished observation). The knock-in approach presented here has cleared this obstacle.

In knock-in transgenic fish, transgenes are integrated in pre-determined sites. This leads to less variability in expression levels of transgenes. Depending on the direction of the integrations (and copy numbers of the transgene, possibly), expression levels can vary (Supplementary Fig. S3), but the variability was much less dramatic than in BAC transgenic fish. With the knock-in, there is little possibility of obtaining lines with very poor expression, unless the expression level of the endogenous gene is extremely low.

### Concluding remark

CRISPR/Cas9 enables the generation of knock-in transgenic zebrafish via a homology independent repair. With the easiness and high efficiency, we propose that this will be a standard technique for the generation of transgenic zebrafish. The same method could be widely applicable for the generation of transgenic animals in other species.

## Methods

### Fish care and strains

Zebrafish adults, embryos, and larvae were maintained at 28.5°C. All experimental protocols were performed in accordance and approved by the animal care and use committees of the National Institutes of Natural Sciences. Animals were staged according to days post fertilization (dpf). Tg[UAS:GFP]^22^, Tg[UAS:RFP]^23^, Tg[BAC-vglut2a-hs:GFP], and Tg[BAC-dbx1b-hs:Cre]^5^ were used in this study.

### Construction of donor DNA for knock-in

Gbait-hsp70:Gal4 was generated by introducing Gbait (GGCGAGGGCGATGCCACCTACGG; this sequence is derived from eGFP) and hsp70 promoter sequence^24^ into the plasmid DNA that has Gal4 (Gal4FF^23^) and BGH (bovine Growth Hormone) polyA sequences^22^. Because the plasmid had a Km resistant gene for BAC (bacterial artificial chromosome) homologous recombination, the resultant Gbait-hs-Gal4 plasmid inherited Km that was located downstream to the BGH polyA. Tbait-hs-lRl-GFPTx and Mbait-hs-lRl-GFPTx were generated as follows. First, the GFPTx fusion gene was constructed by ligating PCR-amplified GFP and Tetanus-toxin light chain^19^. The GFP gene in the hs-lRl-GFP plasmid^5^ was replaced by GFPTx, resulting in the hs-lRl-GFPTx. Tbait- and Mbait-hs-lRl-GFPTx were then generated by inserting Tbait (GGCTGCTGTCAGGGAGCTCATGG) and Mbait (GGCTGCTGCGGTTCCAGAGGTGG) sequence into the hs:lRl-GFPTx plasmid, respectively. The starting plasmid, hs-lRl-GFP, contained Km for BAC homologous recombination^5^, and thus, the Tbait- and Mbait-hs-lRl-GFPTx plasmids inherited Km that was located within the lRl cassette. The Mbait-hs-lRl-ChR was generated from Mbait-hs-lRl-GFPTx by replacing GFPTx with ChR(wide-receiver)-EYFP^22^,^25^. The Mbait-hs-lRl-GFPTx-truncate was a self-ligation product of KpnI-digested Mbait-hs-lRl-GFPTx. This was a mistake product while constructing Mbait-hs-lRl-GFP. We performed injections with this mistake product without noticing this construction mistake.

### Construction of guide RNA vectors

pDR274^10^ was used as the vector for sgRNA generation. Two synthetic oligonucleotide DNAs, 5′-tagg-N_18_ and 5′-aaac-N_18_ (complementary), were annealed, and inserted in the BsaI site of the vector. With the resultant plasmid, sgRNA of GG-N_18_ will be generated by T7 RNA polymerase. DNA sequences for sgRNA are shown in Supplementary Figures S1 and S2. The plasmid DNA for the production of sgG was obtained from Del Bene^15^.

### Preparation of sgRNAs, Cas9 mRNA

Template DNA for sgRNAs was digested with DraI, and sgRNAs were transcribed using the MAXIscript T7 kit (Life Technologies). pCS2-hSpCas9 (a gift from M. Kinoshita and F. Zhang^9^) was digested with NotI, and Cas9 mRNA was transcribed using the mMESSAGE mMACHINE SP6 kit (Life Technologies). sgRNAs and Cas9 mRNA were purified using the RNeasy Mini kit (Qiagen).

### Microinjection

sgRNAs and Cas9 mRNA were co-injected into one-cell stage zebrafish embryos with Qiagen miniprep (Qiagen) purified donor DNA. Each embryo was injected with the solution containing ~6 ng/μl of each sgRNA (one for digesting genomic DNA and the other for digesting donor plasmid), ~130 ng/μl of Cas9 mRNA, and ~6 ng/μl of donor plasmid. In one injection session, ~70 embryos were injected with each injection solution. In our experimental condition, ~50 embryos typically survived. Fluorescent protein expression was monitored at 3 dpf. If we found embryos that had broad RFP expression in the expected region of the body among ~50 embryos, the corresponding sgRNA was considered as a good sgRNA, and it was used for the generation of transgenic fish. If more than one sgRNA for each gene produced positive results, the sgRNA that gave rise to the best expressions was chosen.

For the injection to investigate the indel inducing activity of sgRNAs, each embryo was injected with the solution containing ~12.5 ng/μl of a sgRNA and ~190 ng/μl of Cas9 mRNA.

### Insertion mapping

For insertion mapping, fluorescent F1 embryos at 3 dpf were collected, and genomic DNA was extracted with standard protocols. PCR reactions were performed using a 5′ primer that was specific to each gene (upstream to the expected insertion site) and a 3′ primer that was specific to the donor plasmid (sequence within the hsp70 promoter for detecting the forward insertion, and sequence within pBluescriptSK for detecting the inverse insertion).

### Detection of indel mutations

Injected embryos (10 to 15 in number) were collected at 1 dpf. Genomic DNA was extracted with standard protocols. The targeted region of the genome was amplified by PCR. The resulting PCR products were subcloned into pMD20-T (Takara) using TA-cloning. The insert of individual colonies was PCR-amplified, and sequenced.

### Immunohistochemistry

Zebrafish embryos were processed for immunohistochemistry using standard protocols. Antibodies used were: rabbit anti-En1 antibody^26^ that recognizes Eng1b in zebrafish^16^,^17^ and guinea-pig anti-Evx2 antibody^5^. Cy5-conjugated secondary antibodies (Jackson ImmunoResearch) were used.

### Imaging

Low magnification images were taken using an MVX10 microscope (Olympus). High magnification images were taken using a Zeiss 510 confocal microscope.

## Author Contributions

Y.K. and S.H. conceived and designed the study, performed the experiments, and wrote the manuscript. Y.H. and A.K. instructed CRISPR/Cas9 system, and gave valuable suggestions to Y.K. and S.H. All authors reviewed the manuscript.

## Supplementary Material

Supplementary Information Supplementary Information
