# Supplementary material for: Efficient generation of knock-in transgenic zebrafish carrying reporter/driver genes by CRISPR/Cas9-mediated genome engineering
Source: Sci Rep. 2014 Oct 8;4:6545. doi: 10.1038/srep06545 (PMC4189020; doi:10.1038/srep06545)
Supplement: Supplementary Information [file srep06545-s1.pdf]

Supplementary Information

Efficient generation of knock-in transgenic zebrafish carrying reporter/driver genes by CRISPR/Cas9-mediated genome engineering

Yukiko Kimura[1], Yu Hisano[2], Atsuo Kawahara[2,3], and Shin-ichi Higashijima[1]

[1]National Institutes of Natural Sciences, Okazaki Institute for Integrative Bioscience, National Institute for Physiological Sciences, Okazaki, Aichi 444-8787, Japan

[2]Laboratory for Cardiovascular Molecular Dynamics, RIKEN Quantitative Biology Center, Furuedai 6-2-3, Suita, Osaka, 565-0874, Japan

[3]Laboratory for Developmental Biology, Center for Medical Education and Sciences, Graduate School of Medical Science, University of Yamanashi, Simigatou 1110, Chuo, Yamanashi, 409-3862, Japan

sgG	GGCGAGGGCGATGCCACCTA
Gbait	GGCGAGGGCGATGCCACCTACGG
sgT	GGCTGCTGTCAGGGAGCTCA
Tbait	GGCTGCTGTCAGGGAGCTCATGG
sgM	GGCTGCTGCGGTTCCAGAGG
Mbait	GGCTGCTGCGGTTCCAGAGGTGG

Figure S1

Bait DNA sequences and DNA sequences for their corresponding sgRNAs

The PAM (protospacer-adjacent motif) sequence is labeled in green. Gbait is the sequence in the GFP coding region [15]. sgG is the DNA sequence for the sgRNA against Gbait. Tbait is the sequence in the mouse Tet1 coding region [13]. sgT is the DNA sequence for the sgRNA against Tbait. Mbait is the sequence in the rat Mc4r coding region [18]. sgM is the DNA sequence for the sgRNA against Mbait.

evx2sg1 GGAACAACCATATTCGCAAC
(genome) GAAACAACCATATTCGCAACGGG (-347 ~ -325)

evx2sg2 GGTCAGAGAGGGAGAGAGAG
(genome) GCTCAGAGAGGGAGAGAGAGAGG (-221 ~ -199)

evx2sg3 GGAGGGAGAGCCAGAACAGA
(genome) AGAGGGAGAGCCAGAACAGAAGG (-197 ~ -175)

eng1bsg1 GGATTATCTGTCTGCAATTT
(genome) GAATTATCTGTCTGCAATTTAGG (-271 ~ -249)

eng1bsg2 GGATCCAGTCCTCATTAGAG
(genome) ACATCCAGTCCTCATTAGAGTGG (-214 ~ -192)

eng1bsg3 GGAGTGGATTTAACATTCTC
(genome) AGAGTGGATTTAACATTCTCCGG (-200 ~ -178)

eng1bsg4 GGCTATTTGCGATGTTATTA
(genome) AGCTATTTGCGATGTTATTAGGG (-337 ~ -315)

eng1bsg5 GGATGTTTCTGGATGGGCAC
(genome) GGATGTTTCTGGATGGGCACTGG (-233 ~ -211; opposite strand)

glyt2sg1 GGGGGGTGATGCATGCAGTG
(genome) CAGGGGTGATGCATGCAGTGTGG (-547 ~ -525)

glyt2sg2 GGGCCAGTGACGCAGGAACG
(genome) GTGCCAGTGACGCAGGAACGTGG (-468 ~ -446)

glyt2sg3 GGGCAAACACTTAGGGAAAT
(genome) ACGCAAACACTTAGGGAAATGGG (-415 ~ -393)

vglut2asg1 GGGGGGAAACACCCCTGATC
(genome) GGGGGGAAACACCCCTGATCCGG (-682 ~ -660)

vglut2asg2 GGCTGGCACAGGACTGGCGG
(genome) GGCTGGCACAGGACTGGCGGCGG (-381 ~ -359)

Figure S2

Figure S2

DNA sequences for sgRNAs against zebrafish genomic sequences

sgRNAs were generated using the pDR274 vector and T7 polymerase. This constrained sgRNAs to have GG sequence at their 5' termini. We did not strictly select target genomic sequence that start with GG, as mismatches near the 5' terminus of sgRNA can be tolerated for Cas9-mediated DNA cleavage [9]. Mismatches at the nucleotide position 1 and/or 2 are labeled in red. The PAM sequence is labeled in green. For each gene, numbering of nucleotides is based on the position from the putative transcription start sites, which were determined by the longest cDNAs available (GenBank#; BC057443 for evx2, BC091669 for eng1b, NM_001009557 for glyt2, and AB183386 for vglut2a). The sgRNA used for the generation of stable transgenic fish for each gene is highlighted in bold.

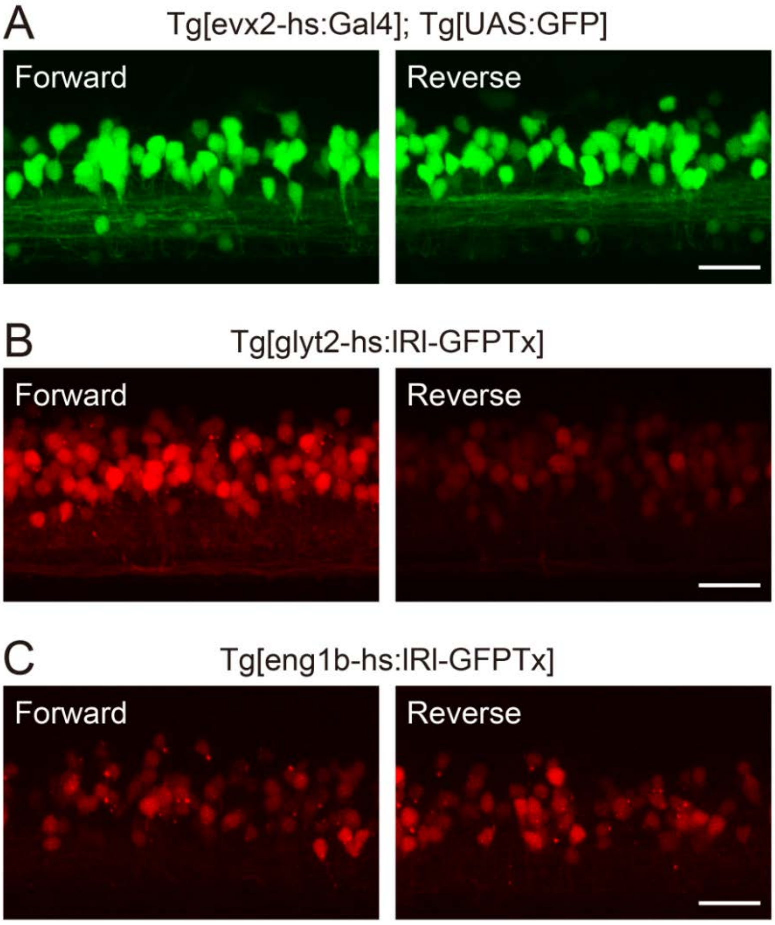

Figure S3

Comparison of transgene expression in forward and reverse insertion lines

All images were lateral views of the spinal cord at 3 dpf. (**A**) Tg[evx2-hs:Gal4]; Tg[UAS:GFP] transgenic fish. The left panel is a fish of the forward integration line, while the right panel is a fish of the reverse integration line. Expression patterns of GFP are similar including fluorescence intensity of GFP. (**B**) Tg[glyt2-hs:lRl-GFPTx] transgenic fish. The left panel is a fish of one of the forward integration lines, while the right panel is a fish of the reverse integration line. Expression patterns of RFP are similar. Fluorescence intensity of RFP in the reverse integration line is weaker. Fluorescent intensities of RFP in the 5 lines of the forward integrations were all similar. (**C**) Tg[eng1b-hs:lRl-GFPTx] transgenic fish. The left panel is a fish of one of the forward integration lines, while the right panel is a fish with the reverse integration. Expression patterns of RFP are similar including fluorescence intensity of RFP. Fluorescence intensities of RFP in the 2 lines of the forward integrations were similar. Scale bars: 20 μm.

**eng1bsg1**	**0% of mutant sequences (0/16)**	
ATACTGAATTATCTGTCTGCAATTTAGGATCTATCTGT	Wt	[x16]

**eng1bsg2**	**0% of mutant sequences (0/16)**	
CAGAAACATCCAGTCCTCATTAGAGTGGATTTAACATT	Wt	[x16]

**eng1bsg3**	**0% of mutant sequences (0/16)**	
TCATTAGAGTGGATTTAACATTCTCCGGCTTATTTGGT	Wt	[x16]

**eng1bsg4**	**0% of mutant sequences (0/16)**	
AAAAGAGCTATTTGCGATGTTATTAGGGTCTAGTTTTG	Wt	[x16]

**eng1bsg5**	**50% of mutant sequences (8/16)**	
TCTGTCAAAGCCAGTGCCCATCCAGAAACATCCAGTCC	Wt	[x8]
TCTGTCAAAGCCAGAAA-CATCCAGAAACATCCAGTCC	-1 (-4, +3)	[x3]
TCTGTCAAAGCC------CATCCAGAAACATCCAGTCC	-6	[x1]
TCTGTCAAAGCCA-------TCCAGAAACATCCAGTCC	-7	[x2]
TCTGTCAAAGCCAG-----------AAACATCCAGTCC	-11	[x1]
TCTGTCAAAGCCAGTGGATGGATGGATGCAGAAACATCCAGAAACATCC AGTCC	+16 (-2, +18)	[x1]

Figure S4

Frequencies and sequences of targeted indel mutations induced by eng1bsg1-5 at the *eng1b* locus

The wild-type (Wt) sequence is shown at the top with the target site in orange and the PAM sequence in green. Deletions are shown as red dashes highlighted in grey and insertions as red letters highlighted in grey. The net change in length caused by each indel mutation is to the right of each sequence (+, insertion; –, deletion). Note that some alterations have both insertions and deletions of sequence and in these instances the alterations are enumerated in the parentheses. The number of times each mutant or non-mutant (Wt) allele was isolated is shown in brackets

evx2sg3 56.3% of mutant sequences (9/16)

GAGAGAGAGGGAGAGCCAGAACAGAAGGAAGGAACAATAGAGA	Wt	[x7]
GAGAGAGAGGGAGAGCCAGA-----AGGAAGGAACAATAGAGA	-5	[x6]
GAGAGAGAGGGAGAG---------AGGGAAGGAACAATAGAGA	-9 (-11, +2)	[x1]
GAGAGAGAGGGAG----------------AGGAACAATAGAGA	-16	[x1]
GAGAGAGAGGG------------------TGGAACAATAGAGA	-18 (-19, +1)	[x1]

glyt2sg2 6.3% of mutant sequences (1/16)

TATATGTGCCAGTGACGCAGGAACGTGGGCGTCAGTTT	Wt	[x15]
TATATGTGCCAGTGACG--------TGGGCGTCAGTTT	-8	[x1]

vglut2asg2 37.5% of mutant sequences (6/16)

CTGCTGGCTGGCACAGGACTGGCGGCGGAGCTCGCGCTGATCTGA	Wt	[x10]
CTGCTGGCTGGCACAGGACTGG----------------------A	-22	[x1]
CTGCTGGCTGGCACAGGACTCCGCGAGCGGCGGAGCTCGCGCTGATCTGA	+5 (-1, +6)	[x1]
CTGCTGGCTGGCACAGGACTG-CGGCGGAGCTCGCGCTGATCTGA	-1	[x1]
CTGCTGGCTGGCACAGGACAGGCACGGCGGAGCTCGCGCTGATCTGA	+2 (-3, +5)	[x1]
CTGCTGGCTGGCACAGGACTGGGCGGCGGAGCTCGCGCTGATCTGA	+1	[x1]
CTGCTGGCTGGCACAGGACTGGACATCATGAACTTTTTCAAGACCGAGAT TACCCTGGCCAACGGCGAGATCCGGAAGCGGCCTCCGGCGGAGCTCGCGC TGATCTGA	+63	[x1]

Figure S5

Frequencies and sequences of targeted indel mutations induced by evx2sg3, glyt2sg2 and vglut2asg2

The wild-type (Wt) sequence is shown at the top with the target site in orange and the PAM sequence in green. Deletions are shown as red dashes highlighted in grey and insertions as red letters highlighted in grey. The net change in length caused by each indel mutation is to the right of each sequence (+, insertion; –, deletion). Note that some alterations have both insertions and deletions of sequence and in these instances the alterations are enumerated in the parentheses. The number of times each mutant or non-mutant (Wt) allele was isolated is shown in brackets.

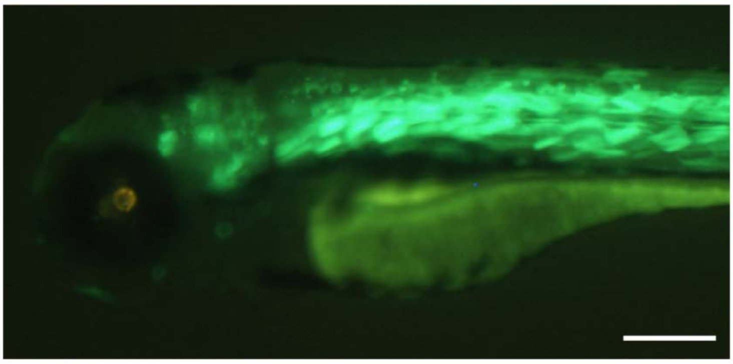

Figure S6

Enhancer trap line

A 3-dpf Tg[ET-hs:Gal4]; Tg[UAS:GFP] embryo. The Tg[ET-hs:Gal4] line was obtained during the course of screening for Tg[eng1b-hs:Gal4]. Scale bar: 200 μm

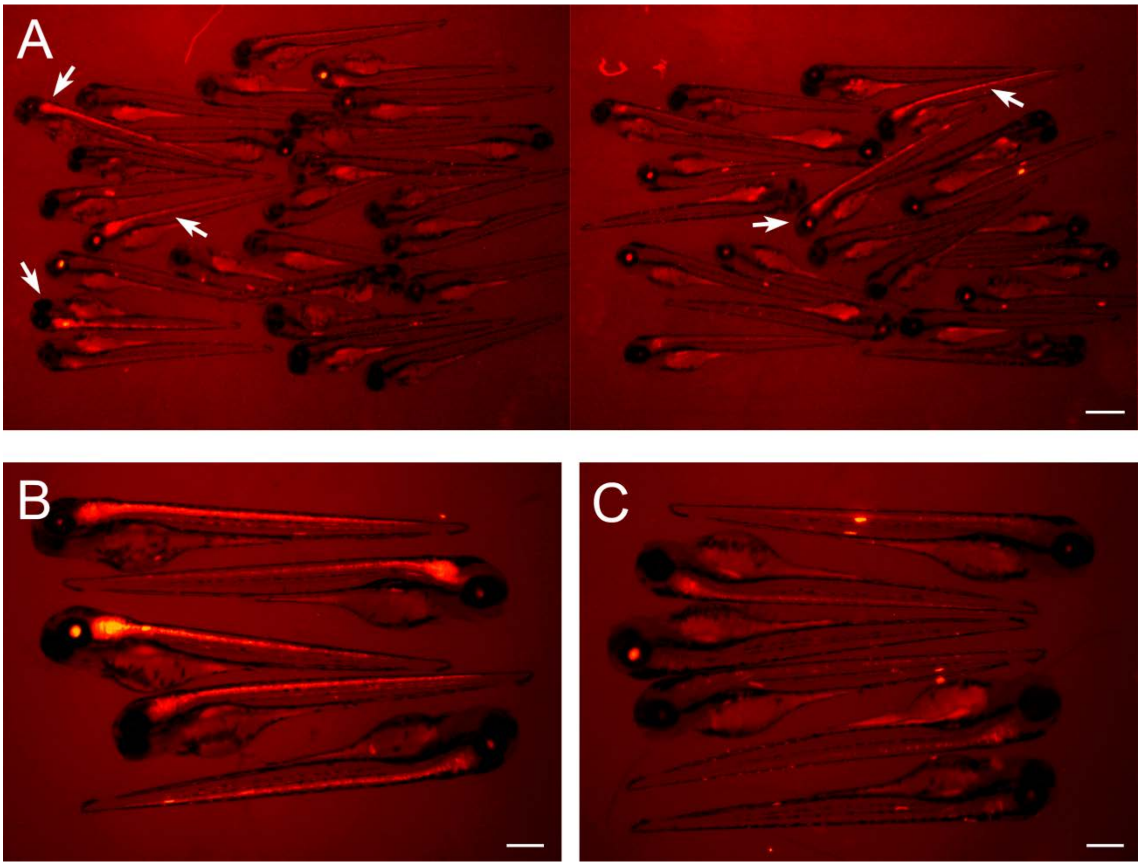

Figure S7

Example of one injection session with a glyt2 construct

Three-dpf embryos showing RFP expression after co-injection of glyt2sg2, sgM, Mbait-hs-lRl-GFPTx plasmid, and Cas9 mRNA. (**A**) All the embryos (approximately 50 in number) in one injection session are shown. The arrows indicate embryos that are shown in (B). (**B**) Five embryos in which RFP expression occurs broadly in the *glyt2* expression domain are shown. These embryos were considered as "good expression". (**C**) Five embryos in which RFP expression occurs in the *glyt2* expression domain are shown. Number of RFP expressing cells in these embryos are not as large as those shown in (B). RFP expression in these embryos was judged to be not sufficient for "good expression". These embryos were grouped into "poor or no expression" together with those embryos that show little or no expression of RFP.

Scale bars: 500 μm in A; 250 μm in B and C.

Transgenic fish	Number of the lines generated	Number of the fish screened	Insertion direction
Tg[evx2-hs:Gal4]	2	17 (no pre-selection)	Forward 1; Reverse 1
Tg[eng1b-hs:Gal4]	1	40 (no pre-selection)	Forward 1
Tg[glyt2-hs:lRl-GFPTx] (Tbait)	4	7	Forward 3; Reverse 1
Tg[glyt2-hs:lRl-GFPTx] (Mbait)	2	3	Forward 2
Tg[glyt2-hs:lRl-GFPTx-truncate]	7	14	Forward 4; Reverse 3
Tg[glyt2-hs:lRl-ChR]	1	4	Forward 1
Tg[vglut2a-hs:lRl-GFPTx] (Mbait)	4	15	Forward 4
Tg[eng1b-hs:lRl-GFPTx] (Mbait)	3	10	Forward 2; Reverse 1

Table S1

Stable transgenic fish generated in this study

It is possible that multiple copies of donor plasmid might have been integrated in some of the transgenic lines. In such cases, the direction of the insertion was only examined for the copy located at the 5' end of the integration.

A

Chromosome	Position	Sequence	Direction	Number of mismatches
11	25965148	GGCTGCaGgCtGGGAGCTCATGG	+	3
17	21971341	GGCTGCaGTCAGtGgGCTCACGG	-	3
8	27848371	GGCTGCTGTCAGtGAGaggAAGG	-	4
18	16878037	GGCgGCTGTtgGGGAGCTCcAGG	+	4
14	8016314	GGCTtgTGTCAGGGAtCTgACGG	-	4
12	8936337	GGCTGCTGTCAGaGctCTCtGGG	+	4
7	48639638	GGCTaCTGTCAGGGgGaTCgCGG	+	4
25	17802164	caCTGCTtTCAtGGAGCTCATGG	+	4
9	31991540	GGgTtCTcTCAGGGtGCTCATGG	-	4
16	14211093	GGtTtCTGTCAGGGtGCaCACGG	-	4
20	33739104	GGtaGaTGTCAGGGAGCTgATGG	+	4
17	16985393	cGCTGCTGTCAGGGAcgTCcGGG	-	4
17	42366669	GGCTGCcGTataGGAGCTCATGG	-	4
15	8592622	GGCTGtTGTCAGGGAGaggACGG	+	4
15	24840731	GGCacCTGTCAGGGAGaTgATGG	+	4
15	31430913	GGCTcCcGTCAGGGAGtTtATGG	-	4
15	44452882	GGtTtCTGTCAGGGtGCaCACGG	+	4
2	36993208	GcCTGCTGgCgtGGAGCTCATGG	-	4
23	21993221	cGCTGCTGTaAGGGccCTCAGGG	-	4
23	44079274	GGCTGCTtaCAGGGAGgaCAAGG	-	4
6	5350438	GGCTGCTGTCAGacAcCTCcAGG	+	4
6	14851243	GGCTGCTGTgAtcGAGCTCcGGG	-	4
6	40171731	GtCTGCTGTaAGGGtGCTgAAGG	-	4
19	9137743	tGCTGCTcTCAaGtAGCTCAAGG	-	4
19	49027213	GGtTtCTGTCAGGGtGCaCACGG	+	4
10	15494713	GGCTGCTGggAGGcAtCTCAAGG	+	4
10	16835202	tGCTGCTGTaAtGGAGgTCATGG	+	4
10	33708343	cGCTGCTGTCAGGGttgTCATGG	+	4
10	44936679	GGCgGCccTCAGGGAGgTCAGGG	-	4
10	44978823	GGCgGCccTCAGGGAGgTCAGGG	-	4
24	43159243	tcCTGCTGTCtGaGAGCTCAGGG	-	4
22	27411451	GGCTGCTGTgAcGGAGgTCtGGG	-	4
4	4546734	GGCTGCaGTgAcaGAGCTCAGGG	-	4
4	10504072	GaCgGCcGTCAGGGAcCTCATGG	-	4
4	11310061	GGCcGCTGTgAGGGgGCTgAGGG	+	4

Table S2A

B

Chromosome	Position	Sequence	Direction	Number of mismatches
3	17728726	GcCTGCTctGGTTCCAGAGGAGG	+	3
14	26895377	aGCTtCTGCGcTTCCAGAGGAGG	+	3
12	15098449	GcCTGtTGtGGTTCCAGAGGAGG	-	3
24	34928049	tGCTGCTGCtGTTCCAGcGGCGG	+	3
22	569116	GcCcGCTGCGGaTCCAGAGGTGG	+	3
8	763047	GGCTGCTGtGGTaCCgGtGGGGG	-	4
8	18470750	aGaTGCTGaGGTaCCAGAGGTGG	+	4
8	55102302	GGCTGCTGCGGgctCtGAGGTGG	+	4
8	55922250	cGgTGCTGCaGTTCCAGcGGTGG	-	4
21	7180499	ctCTGtTGCGGTgCCAGAGGGGG	-	4
21	35407207	GGCaGtgGtGGTTCCAGAGGTGG	+	4
21	35393816	GGCaGtgGtGGTTCCAGAGGTGG	+	4
18	15787324	GGCTGCTGCGGcTgCAGcaGAGG	-	4
18	46986509	GGCTGCTGCaGaaCCAGAaGGGG	+	4
14	21710775	GGCTcCTGaGGTgCCAGcGGAGG	+	4
14	28967438	GGacGCaGtGGTTCCAGAGGTGG	-	4
12	27854317	cGCTaCTGCaGTTgCAGAGGGGG	+	4
7	643190	GGCTGCTtCaGcTtCAGAGGTGG	-	4
7	6239802	GGCTGCaGCaccTCCAGAGGCGG	+	4
7	9168434	GagTGCTGgGGTTCCAGcGGTGG	+	4
7	51715867	tGCTGCaGCtGTTCCAGAcGAGG	-	4
25	31194366	tGCTGCgGCtGTTCCAGAcGAGG	+	4
16	28987146	tGgTGCTGCGGTTCCtcAGGGGG	+	4
16	58244228	GGCTGCTGCGGcTCCtctGGAGG	+	4
20	2770723	GGCcGCgGCaGTgCCAGAGGTGG	+	4
20	13720882	GGCTGCTaCGaTTtCAGAGcGGG	-	4
17	394098	tGCTGCTGCtGTgCCAGtGGAGG	-	4
17	18221938	tGCcGCTGCGcTTCCAtAGGTGG	+	4
15	3394258	GGCTGCTGCGGcTgCgGcGGCGG	-	4
15	10656169	tGtTGCTGCGGccCCAGAGGCGG	-	4
15	32677492	GGaTGCTGCGtTTgCAGAtGGGG	+	4
23	31020962	GGCaGCTGCtGagCCAGAGGTGG	-	4
5	34965582	GaCTGCaGCaGTTtCAGAGGAGG	-	4
6	58794512	GGCTGCTGgGGgTCtAGAGtAGG	+	4
19	3690370	aGCTGCTGCtGTgCCAGAGcAGG	+	4
19	43257174	GGCaGCaGCaGTTCCAGcGGCGG	+	4
13	21664163	aGCTGCTGaGGTTCaAGAGcTGG	+	4
13	50982811	GaCTGCTGtGGTcCCAGtGGTGG	-	4
10	11756781	GGCTGCTGgGGTTCtgGAtGTGG	-	4
24	6073672	tGCTtCTGCGtTTCCAGtGGCGG	+	4
24	18806248	GGCTGtTGaGGTTgCAGAtGTGG	+	4
24	40489046	GGCTGgTGCGGTaCCAaAGaAGG	-	4
22	4728633	tGCTGCgGCGGcTgCAGAGGCGG	+	4
22	29636454	GcCTGCTGCGGgTCCAGccGAGG	+	4
22	32772315	GtCTGCTGCGGgTCCAGccGAGG	+	4
4	61756361	GcCTGCTGCGGgTCCAGccGAGG	+	4
4	61722844	GcCTGCTGCGGgTCCAGccGAGG	+	4

Table S2B

Table S2

Genomic location of potential off-target sites of sgT and sgM

Genomic locations of potential off-target sites of sgT and sgM were identified by using the Cas-OFFinder algorithm (Bae et al., 2014, Bioinformatics 30, 1473-1475). We identified no potential off-target binding sites of sgM and sgT in the zebrafish genome with up to two mismatches. Mismatches are shown in small letters. (**A**) Genomic location of potential off-target sites of sgT. Two sequences showed three mismatches and a conserved PAM sequence (5'-NGG) and 33 sequences four mismatches and a conserved PAM sequence compared to the sgT sequence. (**B**) Genomic location of potential off-target sites of sgM. Five sequences showed three mismatches and a conserved PAM sequence and 42 sequences four mismatches and a conserved PAM sequence compared to the sgM sequence.
